# Supplementary material for: A Long-Term Study on Chemical Compounds and Their Location in Sweet Basil Leaves from Organic and Conventional Producers
Source: Foods. 2024 Jan 24;13(3):383. doi: 10.3390/foods13030383 (PMC10855304; doi:10.3390/foods13030383)
Supplement: Supplementary file 1 [file foods-13-00383-s001.zip › foods-2826800-supplementary.pdf]

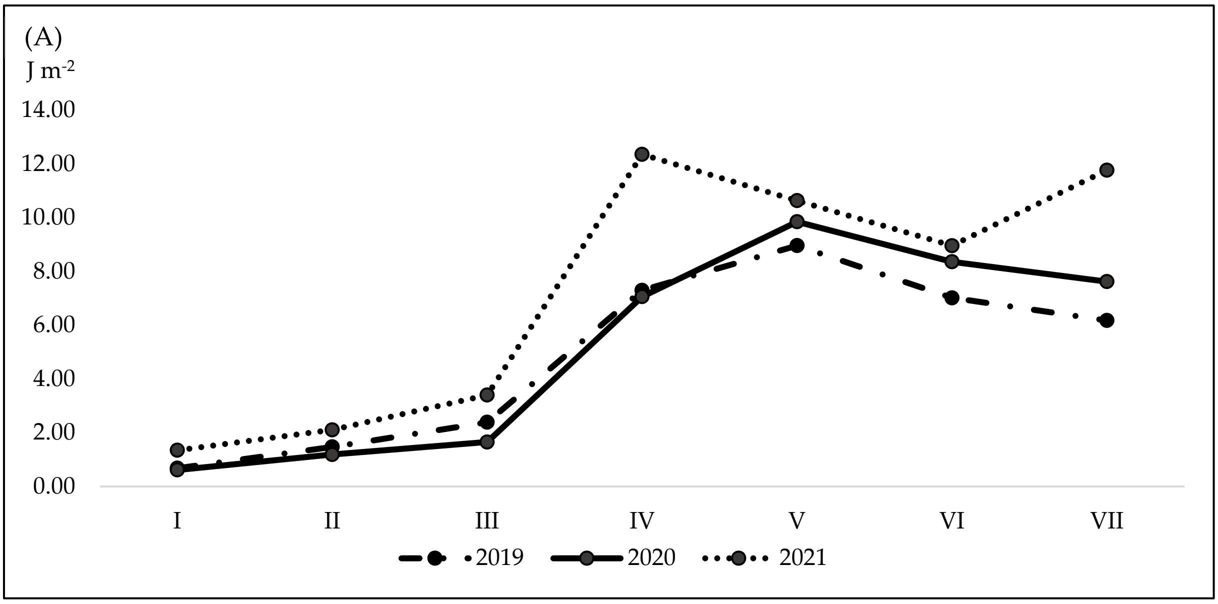


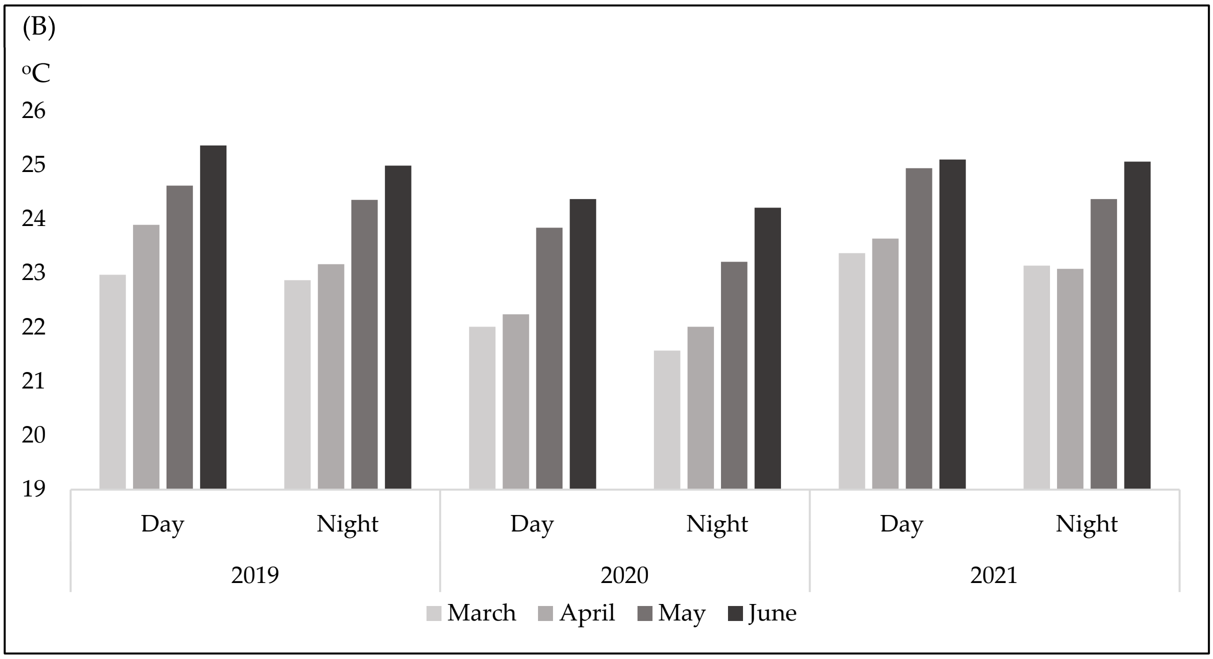


Figure S1. Average monthly sum of radiation (A) and day-night air temperature (B) during basil cultivation (2019-2020).

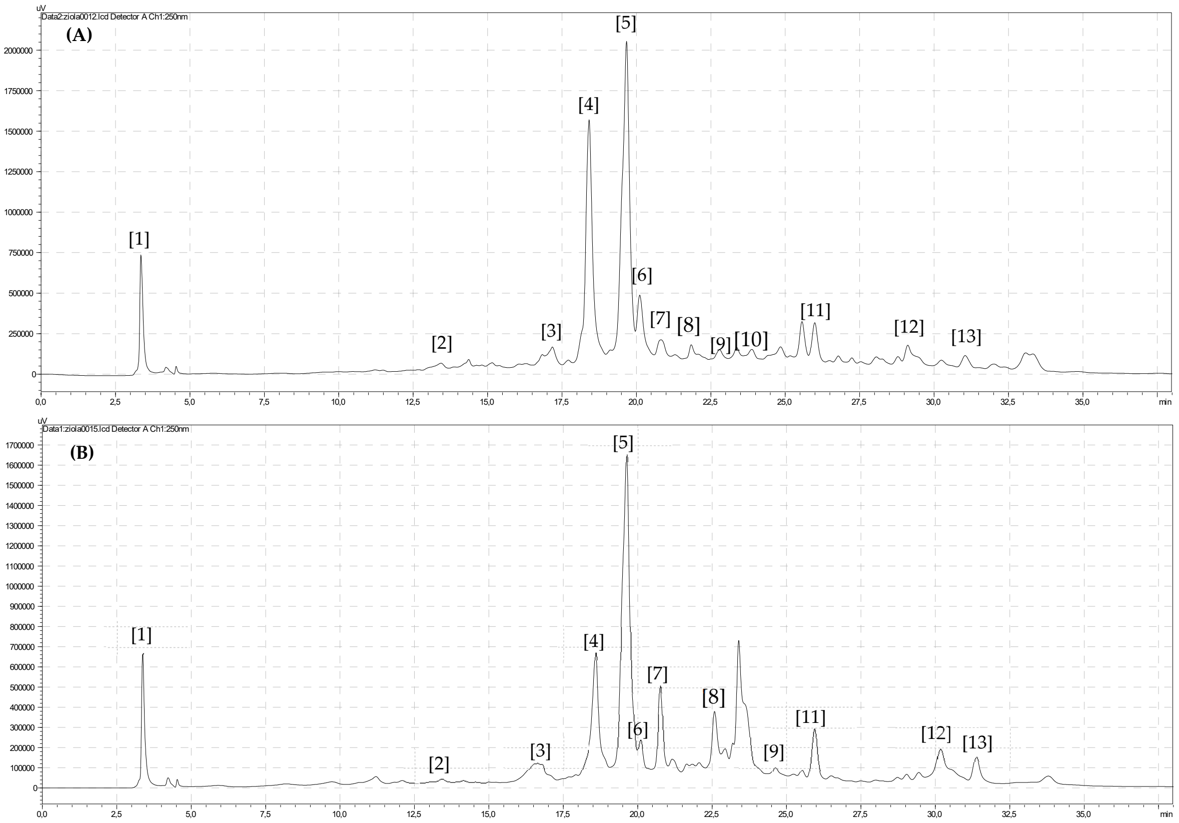


Figure S2. Chromatogram from identification of polyphenols in (A) organic and (B) conventional basil: [1] gallic acid, [2] benzoic acid, [3] ferulic acid, [4] p-coumaric acid, [5] rosmarinic acid, [6] caffeic acid, [7] p-hydroxybenzoic acid, [8] kaempferol-3-O-glucoside, [9] kaempferol, [10] quercetin, [11] quercetin-3-O-glucoside, [12] myricetin, [13] luteolin.

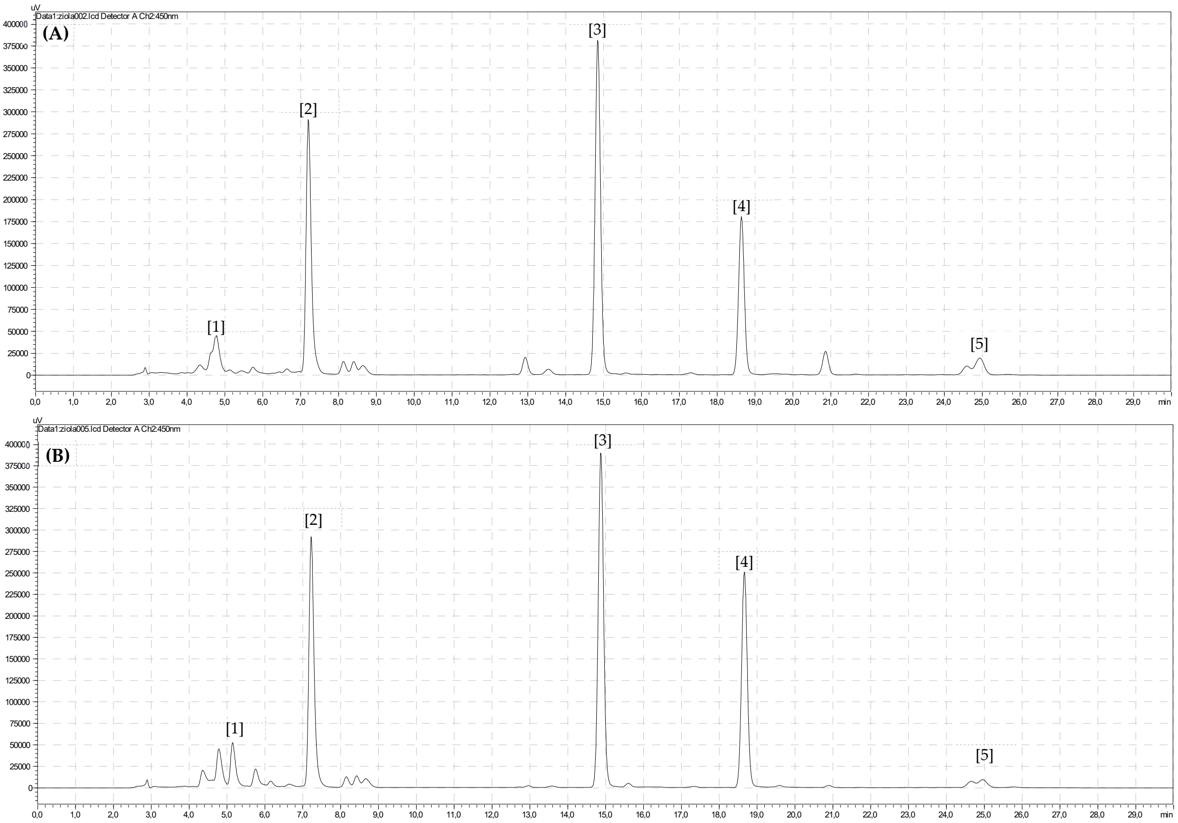


Figure S3. Chromatogram from identification of chlorophylls and carotenoids in (A) organic and (B) conventional basil: [1] zeaxanthin, [2] lutein, [3] chlorophyll b, [4] chlorophyll a, [5] beta-carotene

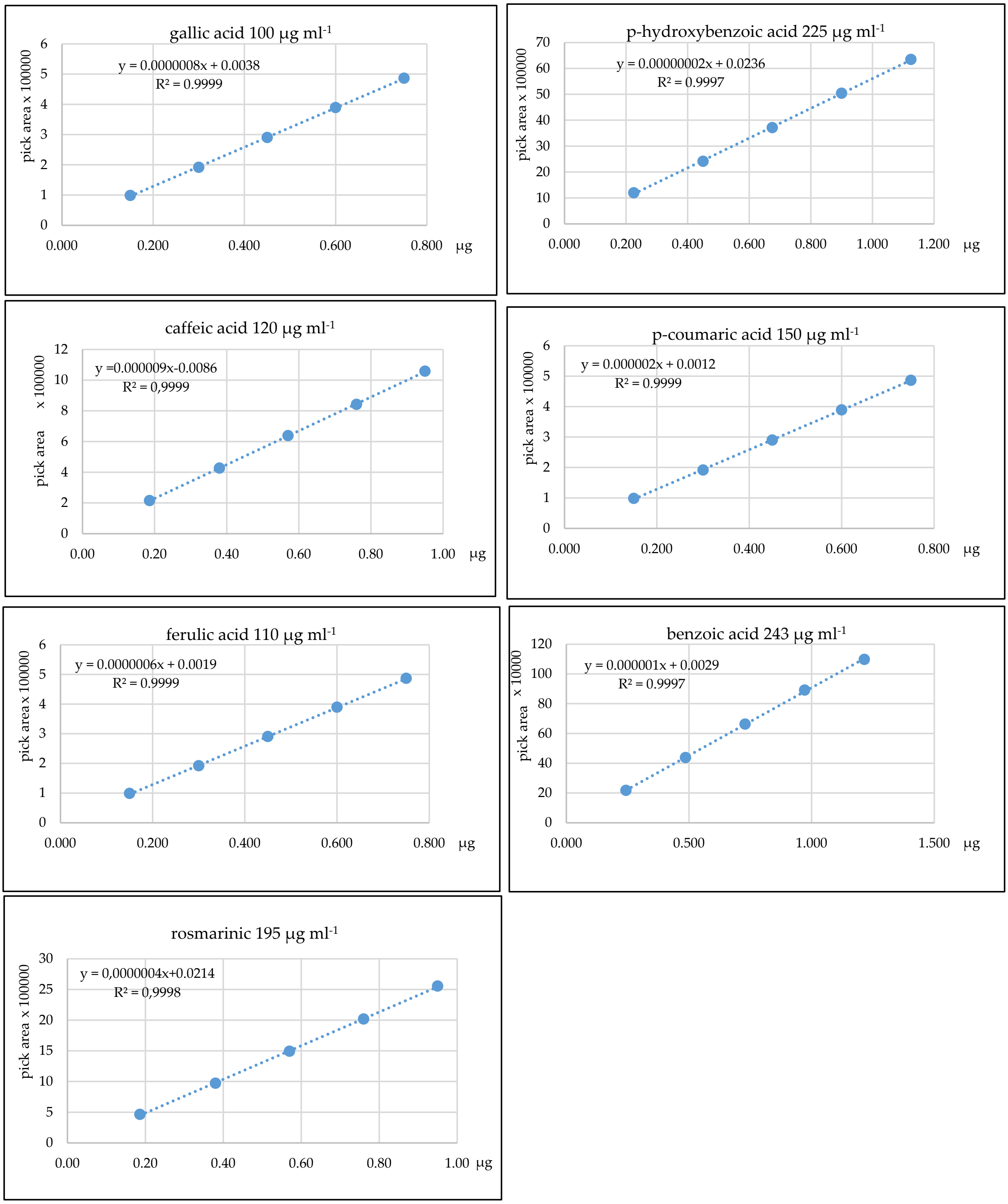


Figure S4. Standard curves for identified phenolic acids in basil leaves

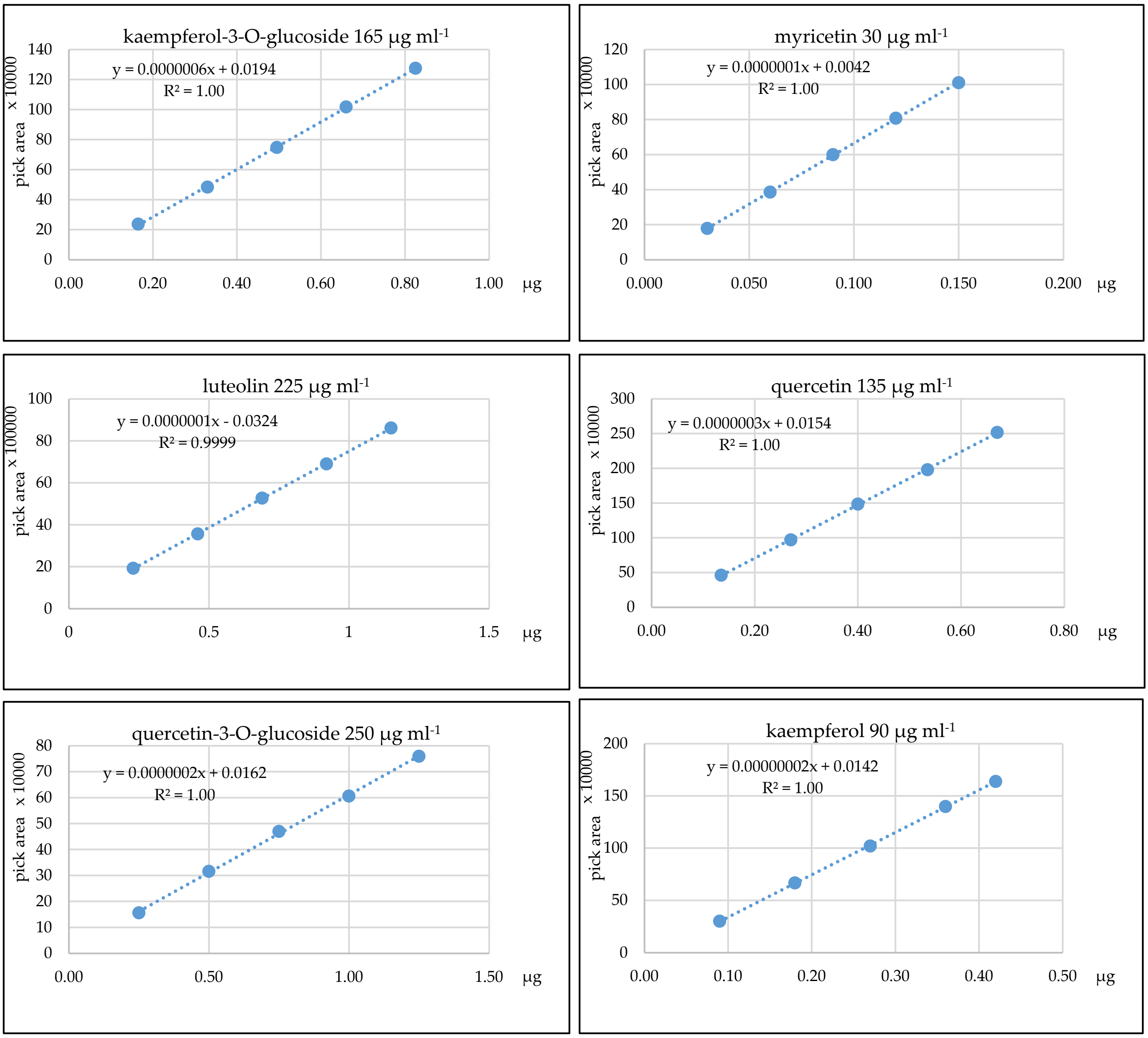


Figure S5. Standard curves for identified flavonoids in basil leaves

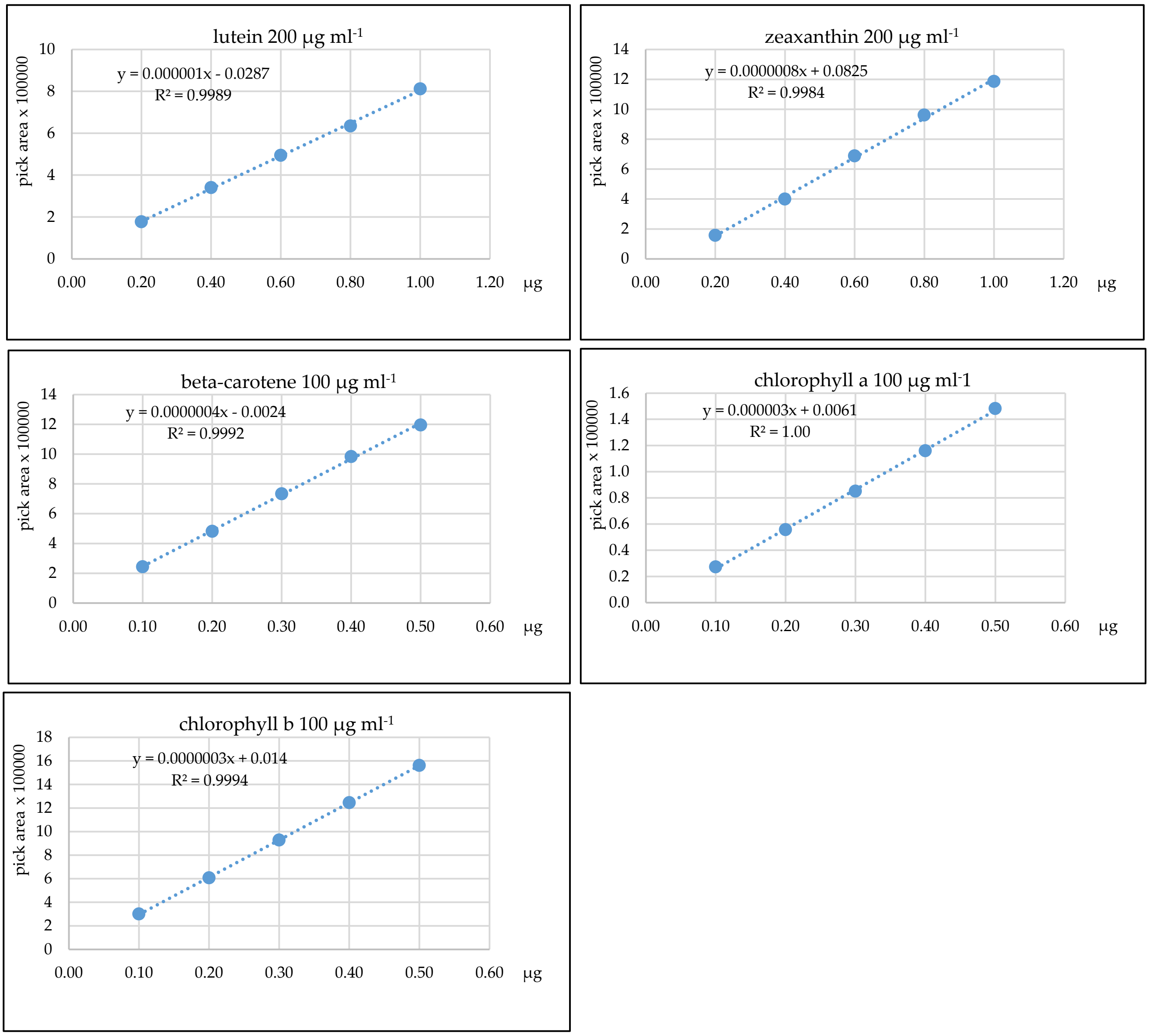


Figure S6. Standard curves for identified carotenoids and chlorophylls in basil leaves

Table S1. Characteristic of fertilizers used by basil producers in time of growth.

| Fertilizer name | fertilizer description | N | P | K | Mg |
|---|---|---|---|---|---|
| Humvit bio | HUMVIT-BIO and Universal is a natural fertilizer that improves the properties of substrates, based on biohumus. This is kind of vermicompost. Natural compost that has been additionally processed by earthworms (*Eisenia fetida*). | 0.030% | 0.030% | 0.040% | - |
| Humvit bio universal | | 0.030% | 0.035% | 0.040% | - |
| Humiplant | HUMIPLANT™ effectively restores the soil's chemical, biological and physical properties making the nutrients in the soil more available for plant uptake. Humic & fulvic acid applied to clay soils breaks up compaction and allows for water penetration. | 0.030% | 0.030% | 0,030% | - |
| Algaplant | Organic biostimulator increasing the absorption of nutrients.<br>This biostimulator contains natural growth hormones that allow the plant to take strong roots. The soil retains nutrients better, so the plant has easier access to them. PURE alga plant 20% is a very concentrated extract of brown algae - the most active and richest in nutrients of seaweed. | - | - | - | - |
| Ziołovit universal | Water extract of guano, which is used to produce fertilizer, is a mixture of seabird droppings, feathers, dead animals, egg shells and sand, | 2% | 2% | 2% | 2% |
| Agrolinija-S | Organic fertilizer (concentrate), using compost of beef cattle manure and organic humus from leonardites using innovative technology. Thanks to this technology, we obtain an organic fertilizer with a very large amount of humic and fulvic acids and NPK macroelements, which gives a striking effect when applied foliarly. | 1.5% | 1.5% | 2.0% | 1.0% |
| Basofoliar 2.0 | Basfoliar® 2.0 6-12-6 is a comprehensive, multi-ingredient, liquid foliar fertilizer with increased phosphorus (P) content. The fertilizer contains all the macroelements necessary for plants: nitrogen (N), phosphorus (P), potassium (K), | 6.0% | 12.0% | 6.0% | - |
